# The Lunatic Asylums and the Medical Colleges

**Published:** 1896-11

**Authors:** F. S. White

**Affiliations:** Terrell, Texas, Late Superintendent Texas State Lunatic Asylum


For the Texas Medical Journal.

### The Lunatic Asylums and the Medical College.

F. S. WHITE, M. D., TERRELL, TEXAS,
Late Superintendent Texas State Lunatic Asylum.

THESE examples of the State's liberality and beneficence to the victims of misfortune on the one hand, and to those being educated and trained to relieve suffering on the other, can be mutually beneficial in the way of increasing the opportunities for study and research of the medical students, and at the same time giving the inmates of the asylums the benefit of nurses of superior qualifications.

Nothing is so conducive to the acquirement of medical knowledge as bed-side training. Newly graduated physicians always feel the want of this sort of information when they leave their *alma mater* and begin the practice of their profession.

Theoretical knowledge is necessary as a foundation, but personal contact with, and observation of sick people are valuable adjuncts to the practical application of these theories. At the bedside a certain insight into disease is gained that cannot be taught by professors or books. A man may graduate with high honors and yet be wholly unprepared to deal with the protean pathological conditions that he is sure to meet early in his career.

There are at least three positions in each of the asylums that could be filled more advantageously to the inmates of the institutions by attentive and interested students than by the average persons who are usually employed for these purposes.

I refer to the positions of apothecary and special nurses in the infirmaries or sick wards. Three of these positions would be open to female students. There are also two other places in each asylum that might be occupied by medical students. These are, the night nurses in the infirmaries or sick wards. I would suggest that these places be set apart by the legislature for internes from the Medical College, each intern to serve one year. The place of apothecary, which pays six hundred dollars per year and all expenses, to be filled from the graduating class. The other places, which pay from three hundred to three hundred and sixty dollars per year and expenses, to be filled from the second-course students. These positions to be awarded upon competitive examinations by the faculty of the Medical College. The interners, of course, would be under the same management and control as other employes of the asylums.

If this plan were adopted, these positions would be prizes that the students would very much desire, and as a result would be a great stimulus to study.

